# Cervical Cancer Treatment and Fertility: What We Know and What We Do

**DOI:** 10.3390/cancers17183057

**Published:** 2025-09-18

**Authors:** Nassir Habib, Salwa Idoubba, Francoise Futcher, Emilio Pieri, Giorgia Schettini, Matteo Giorgi, Ramon Rovira Negre, Centini Gabriele

**Affiliations:** 1 Department of Obstetrics and Gynecology, Francois Quesnay Hospital, 2 Boulevard de Sully, 78201 Mantes-la-Jolie, France; nassir.habib@ght-yvelinesnord.fr (N.H.); salwa_idoubba@um5.ac.ma (S.I.); 2 Department of Obstetrics and Gynecology, Pau Hospital, 4 Boulevard Hauterive, 64800 Pau, France; francoise.futcher@ch-pau.fr; 3 Department of Molecular and Developmental Medicine, Obstetrics and Gynecological Clinic, University of Siena, 53,100 Siena, Italy; pieri9@student.unisi.it (E.P.); g.schettini1@student.unisi.it (G.S.); 4 Department of Surgical Sciences, Gynecological Unit, Valdarno Hospital, 52,025 Montevarchi, Italy; matteo.giorgi@uslsudest.toscana.it; 5 Department of Gynecologic Oncology, Hospital De La Santa Creu I De Sant Pau, 08025 Barcelona, Spain; rroviran@santpau.cat

**Keywords:** cervical cancer, fertility preservation, early-stage cervical cancer, conservative surgery, radical trachelectomy, neoadjuvant chemotherapy, ovarian transposition

## Abstract

Cervical cancer is a major health issue worldwide, with approximately 660,000 new cases a year, particularly in women of reproductive age (47.4 ± 12.8 years at diagnosis). Current advances in screening and immunization have shifted cervical cancer diagnoses to earlier stages; as a result, fertility preservation is an essential component of building a treatment plan. *Objectives:* This systematic review aims to synthesize the existing techniques for fertility preservation with a focus on early-stage cervical cancer (cancer stage IA1-IB1). We will describe the different surgical and medical approaches for the treatment of cervical cancer, followed by an analysis of their oncologic safety and the associated reproductive risks and outcomes. *Methods:* A descriptive synthesis of the strategies for surgical management, including conization, radical trachelectomy, neoadjuvant chemotherapy (NACT), and radiotherapy, was completed. Fertility and successful pregnancy rely on patient selection, prognostic variables, and obstetric outcomes. The use of transposition of the ovaries and cryopreservation in the context of gonadotoxic treatment plans also requires investigation. *Results:* For patients meeting conservative eligibility criteria, conservative surgery for tumors up to 2 cm has been considered a safe oncological management strategy, although evidence remains limited. Pregnancy rate after conization ranged from 36 to 55% and 10 to 38% after radical trachelectomy. Ovarian function can be successfully preserved in >60% of laparoscopic transposition cases but resulted in a less than 15% chance of natural conception; the need for assistive reproductive techniques was often required. *Conclusions:* Fertility-preserving management of cervical cancer is safe and feasible in carefully selected patients, with oncologic outcomes comparable to more radical management. Continued innovation and randomized control trials in treatment paths and oncologic and fertility outcomes will benefit the field.

## Simple Summary

Cervical cancer frequently manifests in women during their reproductive age, representing a health concern in this population. With improvements in early detection and vaccination, more women are being diagnosed at a stage where treatment can be less aggressive. This opens the door to treatments that not only fight cancer but also protect a woman’s ability to have children in the future. Many studies suggest that, in carefully selected patients, effective cancer treatment can be achieved with fertility-sparing approaches, preserving the possibility of a future pregnancy. These strategies may help women keep more options open when facing a cervical cancer diagnosis and guide doctors in making more personalized treatment plans.

## 1. Background

Cervical cancer is the *fourth most common cancer* among women worldwide, with approximately *662,044 new cases and 348,709 deaths* reported in 2022 [1].

In France, it is the *11th most common cancer*, with an age-standardized incidence rate (ASR) of 6.1 per 100,000 women, with approximately 2900 new cases and 1100 deaths annually [2,3].

What differentiates cervical cancer from other types of gynecological cancers is that it directly impacts a reproductive organ, which raises significant fertility concerns, particularly in young women.

Diagnosis usually occurs at an early stage (tumor less than 2 cm) because the majority of cervix cancers are diagnosed through well-organized screening programs [4]. Therefore, survival is very high for early-stage cervical cancer. However, treatments, even though oncologically appropriate, often result in a permanent significant loss of reproductive potential.

A recent study reported that the mean age of diagnosis was 47.4 ± 12.8 years, which leaves a considerable number of patients diagnosed with this condition of potential childbearing age [5].

Cervical cancer is caused by persistent infection from high-risk genotypes of the human papillomavirus (HPV), which is responsible for approximately 99.7% of cervical cancer [6].

HPV types 16 and 18 are considered to be the most oncogenic types, and types 6 and 11 are for benign lesions. Other intermediate-risk oncogenic HPV types (31, 33, 35, and 51) are detected in precancerous lesions of the anogenital tract [6,7].

In seven studies, cervical cancer mortality was reduced by 41% to 92% among women who participated in organized screening compared to non-attenders. Mortality reductions were observed in Western Europe (45–92%) and Northern Europe (41–87%) [8].

The natural history of cervical cancer is well defined. After a precancerous intraepithelial stage, cervical cancer may involve the cervical stroma, invade lymphatic channels, or reach the parametrium, pelvic lymph nodes, or upper wall of the vagina [9].

Involvement of the uterine corpus is rare. These defining characteristics of early cervical cancer, in conjunction with the increasing sophistication of imaging, surgical techniques, and technologies, mean that new strategies are available to facilitate curative treatment options that preserve reproductive potential for selected patients.

International guidelines recommend that all patients of reproductive age are counselled at the time of their cancer treatment regarding the effects this treatment can have on their fertility, and, when available, options for preservation are offered [4,10].

There are also potentially relevant fertility preservation options such as oocyte and embryo cryopreservation, ovarian tissue cryopreservation, oophorectomy, and administering pharmacological gonadal protection, although some of these options are still emerging [11].

Recent developments in cervical screening that includes HPV testing and HPV vaccination have impacted the nature of the risk presented to women. Primary HPV testing presents a 40% better detection of high-grade lesions than cytology alone [12], and the uptake of single-dose HPV vaccinations have led to a decrease in high-grade cervical lesions by 51% in 15–19 year old girls over 5–9 years [13]. Cervical cancer screening has been among the most successful public health strategies in industrialized nations, leading to reductions in cervical cancer incidence of up to 80% in areas with high screening coverage and adequate follow-up for women with abnormal results [8].

Over the past twenty years, the demographic of patients with cervical cancer and clinical profile has shifted, with a cohort of women being diagnosed at under 40 years. Therefore, fertility preservation has become a significant focus area for gynecological oncologists [14].

Unfortunately, worldwide, two out of three women aged 30 to 49 have never been screened for cervical cancer. In high-income countries, 84% of women have been screened at least once in their lifetime, compared to only 9% in low-income countries. This disparity has worsened over time. Additionally, the COVID-19 pandemic may have further exacerbated existing inequalities in screening coverage, as it temporarily disrupted cervical cancer prevention programs [15].

## 2. Cervical Cancer Treatment

The optimal management of cervical cancer depends primarily on the tumor stage and its biological aggressiveness. Standard therapeutic options include surgical resection, chemotherapy, and radiotherapy. In recent years, fertility-preserving surgical strategies have gained increasing attention, particularly in patients with early-stage disease who desire future childbearing.

The 2018 FIGO staging provides the following classification:

***Stage I*:** Carcinoma is strictly confined to the cervix.

- **IA:** Invasive carcinoma that can only be diagnosed microscopically, with a depth of stromal invasion < 5 mm.
  ○ IA1: Stromal invasion < 3 mm in depth.
  ○ IA2: Stromal invasion > 3 mm but <5 mm in depth.
- **IB:** Invasive carcinoma with stromal invasion > 5 mm (greater than stage IA), lesion still limited to the uterine cervix.
  ○ IB1: Invasive carcinoma with stromal invasion > 5 mm and maximum tumor diameter < 2 cm.
  ○ IB2: Invasive carcinoma with tumor diameter > 2 cm and <4 cm.
  ○ IB3: Invasive carcinoma with tumor diameter > 4 cm.

Fertility-sparing surgical options are generally considered for cancers up to FIGO IB1 (tumor diameter ≤ 2 cm) in patients desiring future childbearing. In these *highly selected* early-stage, low-risk cases (typically tumor ≤ 2 cm with limited stromal invasion and no high-risk features), treatment can often be tailored to preserve the uterus [16,17].

Fertility-sparing surgery is not recommended for rare, aggressive cervical cancer histologic subtypes (such as small-cell neuroendocrine, HPV-independent adenocarcinoma, and glassy-cell carcinoma) because these variants carry a high risk of recurrence and poor prognosis [18,19].

A crucial prerequisite for any fertility-sparing approach is rigorous nodal evaluation [19]. While LVSI alone is no longer considered a strict contraindication [20,21], lymph node involvement remains a major prognostic factor and must be carefully ruled out.

Occult lymph node metastases, even in early cervical cancer, contraindicate uterine preservation due to the need for chemoradiation in such cases. Therefore, pelvic lymph node assessment is performed as part of the fertility-sparing strategy. Sentinel lymph node (SLN) biopsy has been incorporated to minimize surgical morbidity; guidelines (NCCN) endorse SLN mapping in stage IA2–IB1 as an alternative to full lymphadenectomy [22]. If an SLN is positive in a frozen section, the fertility-sparing procedure is typically aborted in favor of definitive therapy. In the absence of nodal metastasis, the patient may proceed with a uterine-sparing resection. Recent studies suggest that omitting a full pelvic dissection when bilateral SLNs are negative does not increase recurrence in early disease [23], though long-term validation is ongoing.

In summary, candidates for fertility preservation must have early-stage disease confined to the cervix (generally FIGO IA or small IB1), tumor characteristics indicating low risk of parametrial spread (small size ≤ 2 cm, superficial stromal invasion ≤ 10 mm, no or minimal LVSI), and no evidence of nodal or extra-cervical disease.

**Figure 1:**
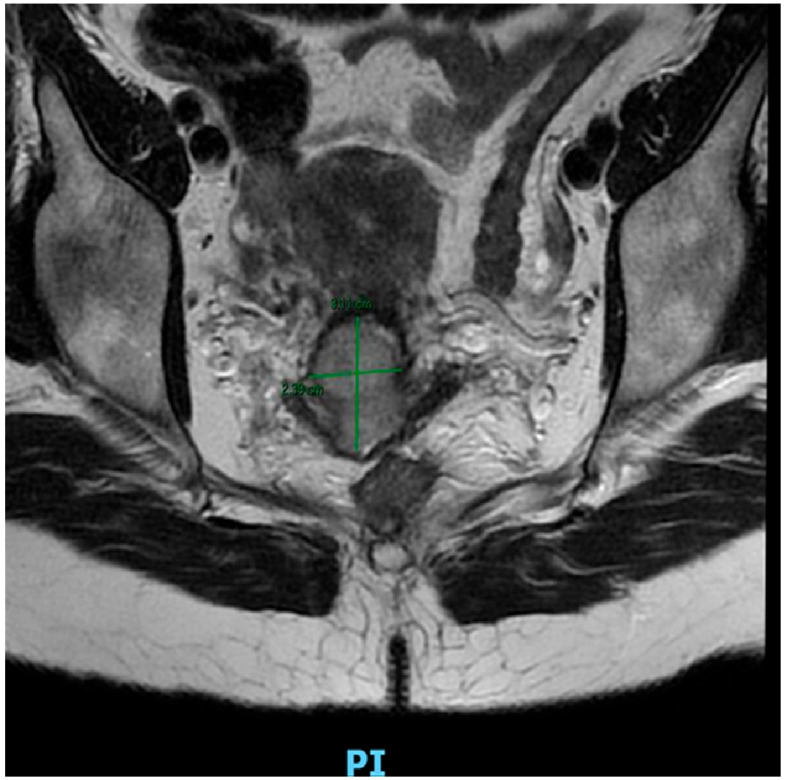
Pelvic MRI (coronal T2-weighted image without contrast) revealing a cervical tumor that measures approximately 3.11 × 2.39 cm, with the lesion appearing hyperintense compared to surrounding cervical stroma, as demonstrated on the images. The lesion is contained to the cervix, which correlates with FIGO stage IB2 cervical carcinoma.

For tumors > 2 cm (FIGO IB2 and beyond), primary fertility-sparing surgery is not standard; in select cases with tumors 2–4 cm, neoadjuvant chemotherapy followed by conservative surgery has been explored, but this remains an investigational approach [16,17] (Figure 1).

## 3. Surgical Techniques

### 3.1. Conization and Simple Trachelectomy

**Surgical Approach and Technique:** According to the European Society of Gynaecological Oncology (ESGO), conization is considered appropriate for stage IA1–T1b1 cervical cancers without lymphovascular space invasion (LVSI) [18]. For stage T1b1 with positive LVSI, however, more radical approaches such as radical trachelectomy are preferred [17].

For patients with stage IA1 and LVSI positivity, conization is typically combined with sentinel lymph node biopsy to assess nodal status.

Cervical conization refers to an excisional biopsy removing a cone-shaped or cylindrical portion of the cervix (including the transformation zone and lesion) using a scalpel (cold-knife cone) or loop electrosurgical technique (LEEP). It is a fertility-preserving excision typically performed for high-grade cervical dysplasia, but in the context of microinvasive cancer it can serve as definitive treatment if certain criteria are met [24].

A *simple trachelectomy* (also called simple cervicectomy) involves surgical removal of the entire cervix (and often a small cuff of the upper vagina) without formal resection of the parametrial tissues, unlike a radical trachelectomy. Simple trachelectomy can be performed vaginally or abdominally and usually includes placement of a cervical cerclage at the uterine isthmus to support future pregnancies. In practice, conization is often used for very early lesions—for example, FIGO IA1 disease without LVSI—whereas simple trachelectomy may be chosen for slightly larger microinvasive tumors or when a prior diagnostic cone had positive margins. The advantages and disadvantages of Vaginal Radical Trachelectomy (VRT) are summarized in Table 1.

**Technical Details:** A cold-knife conization is typically performed under anesthesia, and a full-thickness cone of the cervix is removed; the specimen is oriented and examined to ensure negative margins and measure invasion depth.

If a simple trachelectomy is performed, it often proceeds via a transvaginal approach: an incision is made in the vaginal fornices, the bladder is reflected off the cervix, and the uterosacral and cardinal ligaments are transected close to the cervix (since wide parametrial resection is unnecessary for low-risk tumors). The cervix is removed entirely, and the uterine corpus is then sutured to the vaginal cuff.

A permanent cerclage is placed at the new cervico-isthmic junction to reduce risk of cervical insufficiency in future pregnancies; if adverse findings such as involved margins or lymphovascular invasion are identified postoperatively, completion treatment (e.g., re-excision or even hysterectomy/chemoradiation) may be recommended.

**Oncologic Outcomes:** For appropriately selected early lesions, conization or simple trachelectomy with node assessment has demonstrated excellent oncologic outcomes comparable to more radical surgery. The rationale for these conservative approaches stems from data showing an extremely low incidence of parametrial spread in small, low-risk tumors [25,26]. The recent prospective ConCerv trial provided high-level evidence: it enrolled patients with FIGO 2009 stage IA2–IB1 tumors ≤ 2 cm (squamous or adenocarcinoma) meeting strict criteria (≤10 mm stromal invasion, no LVSI, negative pre-op imaging) and treated them with conization (or simple hysterectomy in those not preserving fertility) plus pelvic lymph node assessment [24]. In the fertility-preservation cohort (44 women), only one patient (2.3%) experienced a recurrence over a median ~2–3 year follow-up [24]. Notably, that recurrence occurred in a case that initially had a 13 mm stromal invasion with positive cone margins, breaching the trial’s inclusion criteria. This underscores the importance of adhering to strict pathologic criteria.

In a prospective study by Schmeler et al. (2021) [24], 44 patients with stage IA2 and IB1 underwent conization with sentinel lymph node dissection as part of a fertility-preserving approach. The recurrence rate in this cohort was only 2.4%.

A large retrospective series by Yang et al. (*n* = 282) analyzed parametrial involvement in stage IB1 disease. Parametrial invasion was observed in 3.7% of tumors < 2 cm, 0.8% in cases with inner one-third stromal invasion, and 3.2% in LVSI-negative tumors, supporting a conservative approach in selected patients [27].

Other series likewise report outstanding disease control. Plante et al. observed a five-year progression-free survival of ~98% after simple vaginal trachelectomy or cone in low-risk early cancers [28].

A multi-institutional review of 347 cases managed by conization found a recurrence rate of only 0.4% [29], highlighting how rare relapse is when stringent selection criteria are met. These data confirm that, in properly chosen patients, conservative surgery does not significantly compromise oncologic safety.

**Reproductive Considerations:** The less tissue removed from the cervix, the better the reproductive outcomes tend to be. Conization (especially a shallow cone) has minimal impact on fertility—most patients can conceive naturally afterward. However, large or repeated cone biopsies may increase the risk of cervical insufficiency and preterm birth. Overall, fertility outcomes after conization/simple trachelectomy are superior to those after radical trachelectomy.

Laser or LEEP (loop electrosurgical excision procedure) conization is generally preferred over cold-knife conization, which has been associated with higher rates of cervical stenosis—14.3% compared to 3.4% for LEEP [29]. Despite an increased risk of preterm birth (*p* = 0.010) and lower birth weight (*p* < 0.001), conization does not significantly increase rates of perinatal death or stillbirth [30,31].

In a systematic review, the pregnancy rate among women attempting to conceive after conservative surgery was ~55%, with a live-born delivery rate around 70% of those pregnancies [32,33].

Another study reported a 36% pregnancy rate among all patients treated with conization [34]. Importantly, because some cervical tissue is retained (or at least no parametrial resection is performed), the incidence of second-trimester losses and extreme prematurity is relatively low in comparison to radical procedures. Still, obstetric management should be alert to the possibility of cervical shortening—serial ultrasounds can be used, and a prophylactic cerclage can be placed in a subsequent pregnancy if indicated by cervical length. In summary, conization and simple trachelectomy offer the *best* fertility and obstetric outcomes of the surgical options, with the trade-off that they are appropriate only for the most favorable, early lesions.

### 3.2. Vaginal Radical Trachelectomy (VRT, Dargent Procedure)

**Patient Selection:** Vaginal Radical Trachelectomy (VRT) is typically offered to women with FIGO stage IA2 or IB1 cervical cancer (tumor diameter up to 2 cm).

Ideal candidates have tumors confined to the cervix without extra-cervical spread, no evidence of lymph node metastasis, and negative lymphovascular space invasion or only minimal focal LVSI. A special caution is taken with adenocarcinomas due to the possibility of skip lesions in the endocervical canal. Tumors should be centrally located and not extensively involving the lower uterine segment, to allow an adequate surgical margin on the uterine side. Generally, a margin of at least 5–10 mm of uninvolved tissue at the uterine end of the specimen is targeted to minimize recurrence risk [35,36].

**Surgical Approach and Technique:** VRT was first described by Daniel Dargent in 1987 as a uterus-sparing alternative to radical hysterectomy. The procedure is performed transvaginally, typically in combination with a laparoscopic lymph node dissection using a modified Schauta technique [37,38]. The surgery usually begins with a laparoscopic evaluation of the pelvis and bilateral pelvic lymphadenectomy (or SLN biopsy), as described by Querleu et al. [39], to confirm absence of nodal disease before proceeding with the trachelectomy. Next, the patient is placed in the lithotomy position for the vaginal phase. A circumferential incision is made in the vaginal fornices around the cervix, and the vaginal epithelium is dissected off the cervix. The bladder is reflected upward (anteriorly) and the rectum downward to expose the parametrial tissues. Under the vaginal approach, the surgeon can resect the parametria (the uterosacral and cardinal ligaments). The uterine arteries are identified and often preserved during VRT [35,36,40]. Dargent’s technique specifically aimed to spare the uterine blood supply when possible, to maintain uteroplacental perfusion for future pregnancies. The cervix, along with a cuff of vagina (usually ~1–2 cm of the upper vagina), and the surrounding parametrial tissue are removed en bloc [41]. This corresponds in extent to a radical trachelectomy, meaning the resection includes similar tissue to a type B2 or C1 radical hysterectomy.

Intraoperative frozen section analysis is routinely performed on the proximal margin (the uterine end) to ensure there is a clear margin of at least 5 mm between the tumor and the cut edge [41,42]. If the margins are positive or too close (< 5 mm) and additional tissue cannot be safely resected (because only a small portion of the lower uterine segment should be taken to preserve fertility), then the procedure is abandoned in favor of a radical hysterectomy to secure oncologic clearance [43]. Assuming margins and nodes are clear, the uterine corpus is then reattached to the vagina. This involves suturing the open end of the vagina to the remaining lower uterine segment. A permanent cerclage is placed around the residual uterine isthmus (sometimes performed just before completing the anastomosis) to act as an artificial cervix, providing support during future gestations. The cerclage effectively mitigates cervical incompetence, since the anatomical cervix has been removed. The vaginal incision is closed, completing the reconstruction. The advantages and disadvantages of Vaginal Radical Trachelectomy (VRT) are summarized in Table 2.

**Oncologic Outcomes:** Vaginal Radical Trachelectomy has demonstrated oncologic safety in numerous studies, with cancer control rates comparable to the traditional radical hysterectomy for appropriately selected small tumors [16,35,44,45]. The recurrence rate following a Dargent procedure has recently been reported to be below 5%, with a mortality rate of approximately 2.5% [46,47,48]. In a five-year follow-up study by Marchiolé et al., the recurrence rate after radical vaginal trachelectomy was 1% for tumors < 2 cm (1/93) and 24% for tumors > 2 cm (6/25) [35].

Early studies by Dargent and others reported very low recurrence rates, establishing proof of concept. More recent long-term data continue to support its efficacy.

For example, a 2024 multi-center study with extended follow-up found VRT to be “oncologically safe”, reporting a recurrence rate on the order of only ~3–5% after many years, with no compromise in survival relative to historical controls [49].

Indeed, multiple retrospective comparisons have shown no significant difference in disease-free or overall survival between radical trachelectomy and radical hysterectomy in tumors ≤ 2 cm [50,51].

In a large systematic review of 2566 patients, the vaginal approach had slightly lower positive margin rates and similar recurrence rates compared to abdominal radical trachelectomy [52].

In a Quebec cohort, the five-year survival rate after fertility-sparing surgery was 95% [46]. About half of the recurrences remain localized within the pelvis—specifically in the parametrium and pelvic wall—while others appear as distant metastases, including intra-abdominal, para-aortic, or supraclavicular lymph node involvement.

Recently, three cases of locoregional recurrence after trachelectomy have been reported. The first involved a pelvic recurrence (at the bladder and iliac lymph nodes) occurring 26 months post-trachelectomy in a patient with a 2.1 cm FIGO IB1 adenocarcinoma and negative lymph nodes, but with only a 5 mm surgical margin [53]. The second case occurred in the rectovaginal and vesicovaginal septum four years post-trachelectomy in a patient with a 1.5 cm squamous lesion (FIGO IB1) and negative nodes, but with lymphovascular space invasion [54]. The third recurrence developed on the cervical stump nearly seven years after surgery for a cervical adenocarcinoma, despite regular six-month follow-ups [55].

It remains unclear whether this case represented a true recurrence or a new primary tumor. It is important to recognize that these favorable outcomes depend on careful patient selection (tumors ≤ 2 cm, node-negative, etc.)—VRT is not intended for tumors beyond those limits, and attempting it in larger or high-risk tumors would likely yield higher failure rates.

Tumor volume appears to be a significant factor in recurrence. According to Dargent, lesions larger than 2 cm are statistically associated with increased recurrence risk [56]. Although lymphovascular space invasion is also a risk factor, it is not an absolute contraindication to trachelectomy, except for high-risk histologies.

**Reproductive Outcomes:** One of the major advantages of the VRT (as initially observed by Dargent) is the favorable fertility outcome relative to more invasive approaches. Among all fertility-sparing options, Vaginal Radical Trachelectomy has shown the highest pregnancy rates—about 67% of those attempting conception were able to achieve pregnancy [57].

Many women resume normal menstrual cycles soon after surgery and can conceive naturally. Once pregnant, however, these patients are considered high-risk obstetrically.

Approximately one-third to one-half of pregnancies after radical trachelectomy will end in miscarriage or preterm delivery due to the absence of a native cervix.

In one long-term series of VRT patients, about 45% of pregnancies ended in premature birth (many in the second trimester) despite the presence of a cerclage [58].

Overall live-born delivery rates per pregnancy are in the range of ~65–80% in experienced centers [59].

Most deliveries are via cesarean section (to avoid stress on the uterine anastomosis and cerclage). Conversely, 75% of patients who reached the third trimester delivered at term by elective cesarean section. The newborns had the expected weight for their gestational age. This aligns with data reported by Klemm et al., who showed via endovaginal Doppler ultrasound that uterine perfusion remains unchanged after vaginal extended trachelectomy [60].

While prophylactic cesarean section is preferred, technically, the cerclage can be cut to allow vaginal delivery if the patient desires. However, due to the reshaped and scarred cervix, dilation may be difficult, increasing the risk of cervical trauma and significant hemorrhage, especially if the tear extends to the uterine pedicles. For patients desiring multiple pregnancies, retaining the cerclage may be advisable. It is reassuring to note that multiple pregnancies are feasible after trachelectomy.

Neonatal outcomes have improved as practitioners gain experience managing these high-risk pregnancies; some centers employ activity restriction, serial ultrasound for cervical length, and even prophylactic hospitalization in the late second trimester to prolong pregnancy.

It is worth noting that the VRT approach appears to preserve fertility slightly better than abdominal or laparoscopic approaches, possibly due to less disruption of pelvic blood supply and less periuterine scarring. Additionally, because VRT patients typically have had no prior abdominal surgery, they avoid potential adhesions that could impact the fallopian tubes or ovaries. In summary, while fertility after any radical trachelectomy is reduced compared to the general population, VRT offers the best odds of conception and carrying a pregnancy closer to term among radical approaches. Counseling is essential: patients should understand the significant risk of prematurity and the need for intensive obstetric surveillance, but also be reassured that a good proportion (over half) of those who attempt pregnancy do succeed in having a live-born child [57].

### 3.3. Abdominal Radical Trachelectomy (ART)

**Patient Selection:** Abdominal radical trachelectomy (ART) is indicated for a similar patient profile as VRT—women with FIGO IA2 or IB1 cervical cancers (generally ≤2 cm) who desire fertility preservation—but it is often chosen in scenarios where a vaginal approach is not suitable, or the surgeon is more comfortable with an open abdominal operation. This could include patients with anatomic or clinical factors making the vaginal route challenging (e.g., narrow vaginal dimensions, prior extensive cervical surgery or scarring, or tumor characteristics that suggest a need for a wider resection).

**Surgical Approach and Technique:** ART was first reported by Smith and colleagues in 1997 as an alternative to the vaginal approach [52]. It involves an open laparotomy (typically a low transverse Pfannenstiel incision) to access the pelvis. The procedure mirrors a standard *radical hysterectomy* (Type C1 radicality) in terms of dissection: the uterus is mobilized, the vesico-uterine fold is opened to drop the bladder, and the uterosacral and cardinal ligaments (parametria) are ligated and divided to remove a wide cuff of tissue around the cervix. The key difference is that, instead of removing the entire uterus, the surgeon amputates the cervix. Generally, the upper one-third of the vagina is also resected en bloc with the cervix and parametria, as in a radical hysterectomy. Pelvic lymph node dissection is performed during the same operation. Some surgeons will identify and *preserve the uterine arteries* during ART to maintain uterine blood flow, while others may sacrifice the uterine arteries as in a typical radical hysterectomy—ovarian artery collateral supply can often sustain the uterus if the uterine arteries are tied. After the cervix, attached parametria, and upper vagina are removed, the remaining uterine body is reattached to the vaginal cuff. This is conducted via an abdominal approach. A permanent cervical cerclage is placed at the level of the new uterine opening.

**Oncologic Outcomes:** Oncologic control with ART appears equivalent to that of radical hysterectomy for early-stage cervical cancer. Because ART allows an extensive parametrial resection under direct visualization, it provides a sense of oncologic assurance for surgeons more accustomed to open surgery. Published series have shown very low recurrence rates. For example, one literature review reported a recurrence rate of ~3.9% after abdominal radical trachelectomy, which is on par with outcomes after radical hysterectomy in similar populations [61,62].

Several retrospective comparisons between ART and VRT have found no significant difference in disease-free survival. In fact, a systematic review encompassing over a thousand ART cases found five-year survival outcomes indistinguishable from historical controls treated with radical hysterectomy [49,50,63].

One advantage of ART is that the surgeon can achieve a wider excision if needed—for instance, a more generous parametrial margin—potentially making ART preferable in cases where tumor size or location is borderline for the vaginal approach. Nonetheless, if a patient does not strictly fulfill the “fertility-sparing criteria” even ART carries a higher risk of relapse, and often alternative treatments (like chemoradiation or neoadjuvant chemotherapy) would be recommended instead.

**Reproductive Outcomes:** Fertility and pregnancy outcomes after ART are less favorable than after VRT. The more invasive nature of ART can impact fertility in several ways: the extensive parametrial dissection and possibly ligation of uterine arteries may reduce uterine perfusion; the open surgery can lead to pelvic adhesions that might involve ovaries or fallopian tubes; and the uterine–vaginal anastomosis creates a fixed opening that could theoretically be more rigid or prone to scarring.

In practice, studies have reported a lower pregnancy rate post-ART compared to VRT. As noted, one review found only ~10% of women became pregnant after ART versus ~38% after VRT [52]. This discrepancy might be partly due to shorter follow-up or differences in patient characteristics, but it likely also reflects the greater disruption caused by an open radical surgery. Nevertheless, those who do conceive after ART can and do have children.

In smaller series, the live-born delivery rates for ART patients who attempt to conceive have ranged from 50 to 70% of pregnancies, not dramatically different from VRT pregnancies, though the absolute number of pregnancies is lower. A recent single-center report of 22 women who underwent ART (including some higher-risk features) noted that 8 patients attempted conception and 5 had successful live-born delivery (62.5% live birth rate among attempters) [64]. The risk of second-trimester loss or preterm delivery remains high after ART, comparable to VRT.

### 3.4. Laparoscopic and Robotic Radical Trachelectomy (LRT/RRT)

**Patient Selection:** Laparoscopic radical trachelectomy (LRT) or robotic radical trachelectomy (RRT) is indicated for the same subset of early-stage cervical cancer patients (FIGO IA2–IB1 ≤ 2 cm, node-negative, fertility desired). In practice, the minimally invasive approach became popular in the 2000s and 2010s as an alternative to open surgery [65], particularly in centers with advanced laparoscopic/robotic expertise. Selection criteria are identical to those for ART in terms of tumor factors. Additionally, the patient’s body habitus and any prior surgeries are considered, as extreme obesity or extensive intra-abdominal adhesions can make a purely MIS approach challenging.

**Surgical Approach and Technique:** MIS radical trachelectomy is essentially the same operation as ART, but performed via either standard laparoscopy or robotic-assisted laparoscopy. The vagina is circumferentially incised from above to detach the cervix and upper vagina. The specimen (cervix with parametria and vaginal cuff) is then removed.

The cerclage placement and reattachment of the uterus to the vagina can be conducted either from above or below. MIS radical trachelectomy avoids a large incision and typically results in lower blood loss (often <200 mL) and shorter hospital stays (often 1–2 days) compared to ART [66,67]. Operative time can be similar to or slightly longer than open surgery, depending on the team’s experience, but contemporary reports show no significant difference in OR time between MIS and open trachelectomy [68].

**Oncologic Outcomes:** The oncologic outcomes of laparoscopic/robotic radical trachelectomy have been generally favorable in early reports, but recent developments have introduced some caution. Initial retrospective studies and meta-analyses suggested that MIS trachelectomy achieved recurrence rates on the order of 4–5%, comparable to open surgery and VRT [32]. 

A large retrospective international study (IRTA study) compared open vs. MIS radical trachelectomy in hundreds of patients and observed no statistically significant difference in recurrence (approximately 4.8% vs. 6.3% at 4.5 years) or overall survival (~99% in both) between the two groups [69].

These data suggest that, in experienced hands and with an appropriate patient selection, laparoscopic or robotic radical trachelectomy does not compromise cancer control. However, the publication of the LACC trial in 2018 showed inferior survival with minimally invasive radical *hysterectomy* for cervical cancer and raised concerns about all minimally invasive approaches in cervical cancer surgery [66]. The LACC trial found higher recurrence and mortality rates in patients who underwent laparoscopic/robotic radical hysterectomy compared to open surgery for FIGO IB1 disease (especially notable in tumors >2 cm).

The limited evidence specifically for radical trachelectomy is more reassuring: as noted, retrospective series like IRTA did *not* show a significant detriment to MIS in the trachelectomy population [69]. Still, because no prospective trial exists (and likely never will, given the rarity of the procedure) to definitively address this, the gynecologic oncology community remains cautious.

**Reproductive Outcomes:** The reproductive performance of laparoscopic/robotic trachelectomy is an evolving story. Earlier reports suggested that fertility outcomes with MIS might be slightly inferior to those with VRT, perhaps due to patient selection biases or unknown factors. In the systematic review by Smith et al., the postoperative pregnancy rate was only ~9% for laparoscopic cases, which was lower than both vaginal (38%) and open abdominal (10%) cohorts [52].

More recent data, particularly with robotic-assisted trachelectomy, paint a more optimistic picture. For example, a large single-center series of 149 women who underwent robotic radical trachelectomy reported a pregnancy rate of 80% among those attempting, with a live-born delivery rate of ~70% [59].

This suggests that in a setting of high expertise, the fertility outcomes can be very good and comparable to the vaginal approach. Robotic surgery may confer some advantages in terms of precision and reduced tissue trauma, which could translate into better preservation of fertility. Additionally, MIS approaches generally cause fewer adhesions in the pelvis, potentially preserving tubo-ovarian function more effectively than an open approach.

Table 3 provides a summary of all fertility-sparing therapeutic options for cervical cancer, organized according to FIGO stage. The surgical techniques corresponding to each approach have been discussed in the preceding sections.

## 4. Neoadjuvant Chemotherapy

Neoadjuvant chemotherapy (NACT) can be offered to patients diagnosed with cervical cancer that have tumors larger than 2 cm, considering a patient’s wishes for fertility-sparing surgery [17]. The motivation for this approach is to reduce tumor size and enable fertility-sparing surgeries. Before starting NACT, pelvic lymph node staging must be completed to ensure that there are no metastases to the lymph nodes [17].

Chemotherapy regimens such as TIP, paclitaxel, ifosfamide, and cisplatin have all been studied in small series for stage IB2 cervical cancer and demonstrate good responses; in most studies, patients were suitable for fertility-preserving surgery. Obstetrical outcomes appear promising; however, these data are derived from small, retrospective studies [70].

There have been several chemotherapy protocols used in the fertility preservation series. The majority of chemotherapy protocols used TIP for squamous cell carcinomas, while paclitaxel-epirubicin-cisplatin (TEP) is utilized for adenocarcinomas. TIP has superior histological responses compared to ifosfamide-cisplatin (IP), with results of histological responses from Lissoni et al. [71] reporting a complete response (CR) rate of 43% and tumor residues less than 3 mm in 25% of cases. TIP has demonstrated greater toxicity, especially hematologic toxicity compared to TP or IP protocols.

At this time, there is no clear consensus on the surgical approach after NACT. According to the literature on the rates of parametrial involvement for tumors 2 cm, the rate of parametrial involvement after NACT for these tumors is unknown.

In early-stage disease, surgeries can be combined with NACT. For locally advanced cervical cancer, combined chemotherapy and radiotherapy is clearly superior to radiotherapy alone. In advanced or metastatic disease, combined chemotherapy is designed to improve overall survival and response rates [72].

## 5. Radiation Therapy

Radiation therapy is a critical aspect in treating cervical cancer, and the recent advancement of this technology makes it easier to target the tumor, thus improving outcomes. The most commonly used radiation therapy is external beam radiation treatment (EBRT). EBRT uses high energy/radiation delivered from the outside of the body toward the tumor or tumor location, along with messages and other healthy tissues close or around the tumor [73].

Intensity-modulated radiation therapy (IMRT) is a variation of EBRT that uses a computer-controlled radiation therapy delivery system with multi-leaf collimeters to change the radiation beam intensity based on the shape of the tumor. IMRT allows for very high doses to be delivered to, or around, the tumor, and significantly decreases radiation to the surrounding healthy structures [74].

Brachytherapy (also called internal radiation therapy) is treatment that uses radioactive sources inserted into or very close to the tumor site. This is used in treatment plans with EBRT to improve treatment efficacy. Intracavitary brachytherapy uses applicators to insert radioactive sources into the vagina and cervix [75], while interstitial brachytherapy directly inserts radioactive sources into the tumor or surrounding tissues using needles.

Image-guided brachytherapy (IGBT) utilizes imaging modalities such as CT or MR imaging before, during, and after placing the applicator to optimize the placement of the applicator and/or plan and deliver the dose distribution. Compared to conventional planning treatment with 2D, 3D planning with IGB treatment improves targeting and reduces toxicity [76].

For locally advanced cervical cancer, definitive treatment comprises EBRT and brachytherapy, plus possibly chemotherapy, to improve treatment efficacy [77]. If there is a likely risk of residual microscopic disease after surgery, postoperative radiation would help reduce the risk of recurrence [78].

Radiotherapy is a well-established and highly successful treatment modality in controlling tumor growth, and this is especially true for cervical cancer, especially when used in combination with chemotherapy and surgery. For example, IMRT and brachytherapy offer patients the chance of preserving organs and decreasing collateral tissue damage, and are options in a patient’s treatments depending on the stage of the cancer and patient characteristics. Likewise, newer technologies, IMRT, and IGBT are constantly improving outcomes [79].

While generally well tolerated, radiation therapy can produce side effects such as skin irritation, pelvic pain, abdominal cramping, diarrhea, and lymphedema [80].

For patients with locally advanced cervical cancer, EBRT, brachytherapy, and chemotherapy are standard treatments [17]. Ovarian transposition is advised during radiotherapy to preserve ovarian function, as the radiation dose to the ovarian cortex is potentially lethal [81].

## 6. Ovarian Transposition

Radiotherapy is well known for its gonadotoxic effects, particularly in young women. Among all organs, the ovaries are especially radiosensitive—exposure to as little as 2 Gy can result in the loss of up to 50% of oocytes.

In the context of cervical cancer, pelvic radiotherapy often delivers cumulative doses between 20 and 30 Gy, making ovarian failure almost unavoidable without protective intervention [82].

This biological vulnerability forms the basis for ovarian transposition (OT), a fertility-preserving surgical strategy designed to reposition the ovaries outside the radiation field. By doing so, OT helps preserve not only hormonal function but also the potential for future fertility. The procedure is especially important given the growing number of young women diagnosed with early or locally advanced cervical cancer who wish to maintain their reproductive potential.

Ovarian transposition (OT) was first described in 1956 by Batten and Brown, who repositioned the ovaries to preserve hormonal function during pelvic radiotherapy in a neuroblastoma patient [83].

Then, in 1958, McCall transposed the ovaries anteriorly to avoid pelvic irradiation [84]. In this pioneering approach, the ovaries were sutured together and fixed to the anterior parietal peritoneum [83]. Since then, OT has evolved from laparotomy-based techniques to more refined laparoscopic and even robotic approaches [85,86].

One advanced technique involves high ovarian transposition without retroperitoneal tunneling of the ovarian vessels. In this approach, the gonadal vessels are mobilized retroperitoneally toward their vascular origin—from the aorta and inferior vena cava on the right, and from the aorta and left renal vein on the left. The ovaries are then lateralized to the upper abdomen while preserving their vascular supply [87].

The procedure is most often performed via laparoscopy, with trocars placed higher than in standard pelvic surgery. According to Moawad et al., techniques include lateral and medial transpositions, with lateral paracolic gutter placement yielding better hormonal outcomes [85]. Care is taken to mobilize the utero-ovarian and infundibulopelvic ligaments while avoiding ureteral injury. In this technique, the fallopian tube is often separated from the ovary, making in vitro fertilization (IVF) necessary for future pregnancy. The ovary is then pulled through the tunnel and fixed to the abdominal wall [88].

However, fallopian tube preservation is feasible and associated with occasional spontaneous pregnancies [45,47].

Successful OT requires meticulous surgical planning, including high lateral positioning of the ovaries—ideally above the pelvic brim—to minimize radiation scatter. Hoekman et al. note that ovarian repositioning to a cranio-lateral site is preferable in pelvic RT [89]. Despite optimal placement, migration or inadequate fixation may still expose ovaries to harmful doses.

Importantly, transposing the ovaries at least 1.5 cm above the iliac crest is associated with a significantly higher chance of maintaining ovarian function after radiotherapy [90].

To ensure ovarian preservation, a small fragment of ovarian tissue may also be excised during surgery and cryopreserved, particularly when the radiation dose is expected to compromise ovarian cortex viability [91]. This procedure is recommended in premenopausal women undergoing pelvic radiotherapy, in order to preserve hormonal function and delay early menopause [92]. Several studies report that menstruation resumes in a substantial proportion of women following ovarian transposition, and hormonal markers such as FSH and estradiol confirm preserved ovarian endocrine activity [92]. Although ovarian transposition preserves hormonal function in most cases, the likelihood of natural conception is often reduced due to disconnection from the fallopian tube. Consequently, many patients require assisted reproductive technologies (ART) to conceive [88].

In a systematic review by Buonomo et al. (2021) [82], ovarian function preservation following ovarian transposition and pelvic radiotherapy (with or without chemotherapy) was achieved in 61.7% of cases. The review included 12 studies, reporting ovarian preservation rates ranging from 16.6% to 100%, with only a 0.4% incidence of ovarian metastases.

Several large reviews have confirmed OT’s effectiveness. A meta-analysis of 24 studies (*n* = 892) reported ovarian function preservation in 90% of women receiving surgery alone, 94% with brachytherapy, and 65% with external beam radiotherapy (EBRT) [93]. Another review on 1189 patients found a 70% overall preservation rate and very low risk of ovarian metastases (1–2%) [86].

According to Hoekman et al. (2019) [89], ovarian function after OT and pelvic radiotherapy was preserved in 20% to 100% of patients, depending on the radiation technique and chemotherapy use. OT combined with brachytherapy alone resulted in the highest preservation rates (63.6–100%), followed by EBRT alone (20–100%) and chemoradiation (0–69.2%).

While effective, OT is not without risks. Complications were reported in 12.8% of patients. These included ovarian cyst formation (27–83%), particularly when ovaries are placed subcutaneously [86,93], abdominal pain (5.4%), and rare cases of ischemia, tubal ligation, or hematoma. Surgical reintervention was required in approximately 35% of these cases. Ovarian metastases occurred in only 0.9% of cervical cancer cases, confirming that OT remains oncologically safe for most patients [89]. OT should always be discussed at diagnosis, ideally before planning RT.

Future directions include the refinement of surgical techniques to minimize the need for IVF, the integration of ovarian tissue cryopreservation as an adjunct to OT, and the development of standardized criteria to better predict which patients will benefit most from this intervention.

## 7. Indication for Closure Treatment

Regarding the so-called “closure” hysterectomy after radiotherapy or RCC (concurrent chemoradiotherapy), the debate no longer exists across the Atlantic following the GOG trial [94], which randomized 256 patients with stage IB2-IV cervical cancer into two groups: exclusive radiotherapy (*n* = 124) and radiotherapy followed by surgery (*n* = 132). This trial found no difference in overall survival. However, hysterectomy reduced the risk of local recurrence, which is known to be particularly challenging for patients.

A study by the French Federation of Cancer Control Centers (FNCLCC) investigated the impact of RCC with brachytherapy on invasive cervical cancers of stage IB2-IV and the so-called “closure” hysterectomy in 175 patients: complete histological response was found in 39% of patients, 11% had microscopic residue, and 50% had a macroscopic residue. Also, hysterectomy was associated with a 28% complication rate (mainly urinary). The five-year recurrence-free survival was 66%, influenced by younger age, positive lymph node status, and the presence of residual disease [95].

Few studies have focused on the role of closure surgery based on therapeutic response. The FNCLCC Gynéco-02 study included 61 patients with stage IB2-II cervical cancer who were randomized in cases of complete therapeutic response after RCC into two groups, with or without surgery, with a median follow-up of 3.8 years (range 0.4–5.8). Although this trial was prematurely terminated due to insufficient patient recruitment, no benefit was found for closure surgery [96]. Two French studies (Gustave Roussy 114, rue Édouard-Vaillant 94,805 Villejuif Cedex—France and Paoli-Calmettes 232 Boulevard Sainte Marguerite BP 156 13,273 Marseille institutes) focused on non-responding patients after RCC. Ten patients with residual tumors larger than 2 cm (mean initial tumor size of 6 cm, stage IB2 in eight cases, and stage II in two cases, with a follow-up of 22 months): the results were described as “disappointing” by the authors, but only one pelvic recurrence was observed in a patient who could not undergo in sano resection [97].

Thirty patients with an initial mean tumor diameter of 6 cm (IB2: 5, II: 10, III: 5, and IV: 10) underwent complete surgery (hysterectomy or pelvic exenteration) after RCC: with a follow-up of 45 months, the authors highlighted a five-year overall survival of 80% in the absence of lombo-aortic involvement and emphasized the benefits of this heavy surgery in terms of local quality of life before metastatic progression [98].

## 8. Discussion

All global teams agree that advanced-stage cervical cancers (stages Ib2, II, III, and IV) require multimodal treatment, which includes, at a minimum, radiotherapy combined with chemopotentiation [17]. Therefore, preserving fertility is not feasible in the treatment of such tumors.

Microinvasive cervical cancers (stage IA1) can be managed conservatively through conization, provided that the conization is performed with clear margins and the patient agrees to regular colposcopic and cytological follow-up.

Early-stage cervical tumors (stages Ia2 and Ib1) can, under certain conditions, be treated in a way that preserves both ovarian function and fertility. These early lesions, which have a favorable prognosis, can be managed with surgery alone [17,99]. Ovarian preservation can even be considered in cases of cervical adenocarcinomas. Indeed, for adenocarcinomas ≤ stage IB1, the risk of ovarian metastasis is less than 2% [99].

With regard to fertility preservation, the indications for radical trachelectomy concern young women (under 40 years) with lesions less than 2 cm without endocervical or regional lymph node involvement who wish to preserve their reproductive potential [100]. For lesions measuring between 2 and 4 cm, the risk of recurrence is not negligible (around 10–15%), and patients must make their decision fully aware of these recurrence risks [101].

In addition, it is imperative that the pre-operative assessment of these early-stage tumors includes an MRI evaluation of the size and extent of the lesion. Surgery such as trachelectomy can only be proposed if the tumor exhibits exocervical growth without isthmic involvement.

Prognostic factors in cervical cancer can be classified into pre-treatment and post-treatment categories. Pre-treatment factors include age less than 40, FIGO stage, tumor size exceeding 4 cm—leading to the creation of the IB2 sub-stage—and the depth of stromal invasion [17]. Lymph node involvement is also critical, particularly the number of affected nodes, their anatomical location, and the presence or absence of capsular rupture. The five-year survival rate is 60% when nearby tissues, organs, or regional lymph nodes are affected, against 91% in early stages. [102] Other significant elements include the presence of metastatic emboli and specific histological subtypes, such as glassy-cell carcinoma and neuroendocrine tumors [103]. Post-treatment factors primarily concern the quality of initial treatment, which depends on the accuracy of the pre-therapeutic assessment and the appropriateness of the chosen therapeutic strategy. The response to concurrent chemoradiotherapy (CCR) is particularly relevant, as surgical salvage in cases of recurrence often yields disappointing outcomes. In clinical practice, three key elements stand out. The FIGO stage influences treatment decisions, with smaller lesions potentially allowing for conservative management, whereas tumors exceeding 4 cm generally require CCR. Lymph node involvement necessitates CCR, and its assessment is essential for defining optimal radiotherapy fields. Additionally, aggressive histological subtypes, such as neuroendocrine tumors, require first-line chemotherapy due to their high malignancy potential.

The recurrence rate following a Dargent procedure has recently been published [46,47,48]. It was below 5%, and the mortality rate was 2.5%. These results are comparable to those reported after performing a radical hysterectomy for neoplasms of the same stage. In a Quebec study, the five-year survival rate after fertility-sparing surgery was 95% [46]. Half of the recurrences remain localized in the pelvis (parametrium and pelvic wall), while others manifest at a distance (intra-abdominal metastases and para-aortic or supraclavicular lymph node metastases). The Toronto team of Covens compared the survival of patients who underwent radical vaginal trachelectomy to that of patients who underwent radical abdominal hysterectomy for a similar lesion. No differences in survival were observed [101].

Recently, three cases of locoregional recurrence have been reported. The first occurred 26 months after trachelectomy and was localized in the pelvis at the vesical and iliac lymph node levels. The initial tumor was a FIGO IB1 adenocarcinoma measuring 2.1 × 2.0 cm. Pelvic lymph nodes (*n* = 30) were all negative, but the surgical margin was only 5 mm [53]. The second recurrence occurred in the rectovaginal and vesicovaginal septum four years after trachelectomy. It was a squamous lesion of FIGO IB1 stage, measuring 1.5 cm, with 14 negative lymph nodes. The surgical margins were greater than 10 mm, but there was vascular space invasion [54]. The third recurrence appeared on the remaining cervix nearly seven years after performing a trachelectomy for cervical adenocarcinoma, despite regular follow-ups every six months [104]. It is unclear whether this is truly a recurrence or a second primary lesion.

The risk of recurrence appears to be correlated with tumor volume. Indeed, Dargent’s data indicate that a lesion larger than 2 cm is statistically associated with a higher risk of recurrence [56]. Lymphovascular space invasion is also a risk factor for recurrence, although it does not constitute an absolute contraindication to the procedure. The histological type, except for particularly aggressive subtypes such as small-cell tumors, does not appear to be associated with the risk of recurrence. Even though it is more frequently localized in the endocervix, adenocarcinoma is not clearly linked to an increased risk of recurrence.

Lymph node status represents the most significant independent prognostic factor for survival and recurrence. The discovery of incidental lymph node metastases radically alters the type of intervention. In fact, the conservative procedure is abandoned in favor of concurrent chemoradiotherapy, with the radiotherapy field limits defined by para-aortic lymph node status. During the intervention, the intraoperative analysis of the sentinel lymph node is the method of choice for detecting lymph node metastases [105,106].

After a conization, there is no significant change in infertility management compared to women who have not undergone such procedures. This treatment does not increase the costs associated with assisted reproductive technologies if needed. In contrast, following a trachelectomy, obstetric management becomes more complex due to a higher rate of cesarean deliveries, leading to an increase in medical costs related to childbirth. For patients requiring pelvic radiotherapy, ovarian transposition may be performed to preserve ovarian function. In such cases, part of the ovary can be cryopreserved to allow for fertility preservation in case of ovarian damage. However, cryopreservation of ovarian tissue involves additional costs, particularly for tissue retrieval, long-term storage, and potential future use in assisted reproduction.

**Figure 2:**
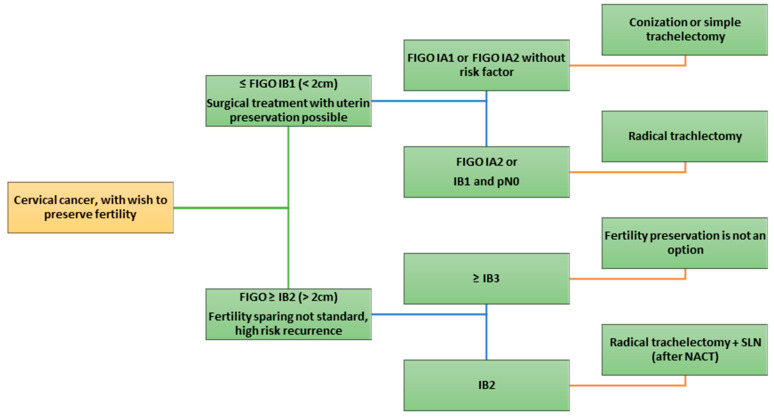
Flow chart on fertility-sparing surgery depending on FIGO stage.

In Figure 2 is shown the flowchart on fertility-sparing surgery depending on FIGO stage.

## 9. Conclusions

Research regarding invasive cervical cancer now consistently supports a personal approach to management. In the early stage, patients show increased potential with treatment involving de-escalation therapy, with anatomical and less radical surgeries that adhere to the principles of fertility sparing. In the advanced stage, we want to improve complete response rates in the primary tumor and the involved lymph nodes.

Emerging concepts such as conformal intensity-modulated radiotherapy (IMRT), post-RCC chemotherapy, and targeted therapies offer new opportunities to provide the best possible outcomes for women with invasive cervical cancer.

## Figures and Tables

**Table 1:** Summary of key advantages and limitations of conization and simple trachelectomy as fertility-sparing procedures in early-stage cervical cancer.

Advantages		Limitations	
**Maximal Fertility Preservation**	Leaves most of the reproductive tract intact. Menstrual and reproductive functions are generally unharmed, and conception rates are high.	**Restricted Indications**	Only suitable for very early-stage cancers with favorable features.
**Minimal Morbidity**	Conization is typically an outpatient or short-stay procedure with quick recovery.	**Need for Careful Pathologic Assessment**	Margins must be clear and lymph nodes free of metastasis. Close coordination with pathology is required.
**Low Complication Risk**	Lower rates of blood loss, infection, and surgical complications.	**Risk of Re-intervention**	If final pathology reveals unexpected adverse features, patients may need a second surgery or adjuvant therapy.
**Oncologic Safety in Low-Risk Cases**	Outcomes are equivalent to radical surgery.	**Cervical Insufficiency**	Removing all or part of the cervix increases the risk of miscarriage or preterm birth.
**Avoidance of Parametrial Resection**	Spares the autonomic nerves and vascular supply that would be removed in a radical procedure and improves postoperative quality of life.	**Surveillance Burden**	Patients must adhere to intensive follow-up. Any sign of recurrence mandates prompt intervention, and the emotional burden of ongoing surveillance can be high.

**Table 2:** Summary of key advantages and limitations of VRT as fertility-sparing procedures in early-stage cervical cancer.

Advantages		Limitations	
**Avoidance of Laparotomy**	All radical resection performed through the vagina.	**Technical Complexity and Expertise**	Technical Complexity and Technical Stewardship: Because VRT is a technically challenging operation, it requires a significant degree of surgical experience in radical pelvic surgery in the vaginal approach.
**Organ Preservation with Radical Oncologic Control**	Patients keep potential for fertility and have regular menstrual function. Survival outcomes are similar to radical hysterectomy for appropriate tumors.	**Tumor Size Limitations**	The vaginal approach is typically only safe for small tumors (≤2 cm).
**Higher Pregnancy Rates**	Compared with abdominal or minimally invasive radical trachelectomy, VRT has more favorable fertility outcomes.	**Need for Combined Approach**	VRT needs a combined approach for lymph node dissection with laparoscopy (or robotically assisted), adding to the complexity of the procedure.
**Lower Surgical Morbidity**	Some studies demonstrated shorter operating time and reduced blood loss with VRT compared to an abdominal approach.	**Conversion Risk**	Intraoperative findings may lead to conversion to non-fertility-sparing procedure.
**Better Obstetric Outcomes**	All radical trachelectomy have obstetric risks, but patients undergoing VRT demonstrate less severe prematurity	**Obstetric Challenges**	Despite being the most fertility-friendly radical option, there is still a significant risk of miscarriage and preterm birth. Each patient is likely to need significant monitoring in pregnancy, which may require even more specialist care.

**Table 3:** Summary table of fertility-sparing surgical options for early-stage cervical cancer according to the FIGO classification. The table describes the tumor characteristics (lymphovascular space invasion, depth of stromal invasion, tumor size), suitable fertility-preserving procedures, and eligibility requirements and surgical considerations. Abbreviations: LVSI = lymphovascular space invasion; LEEP = loop electrosurgical excision procedure; SLN = sentinel lymph node; PLND = pelvic lymph node dissection; MIS = minimally invasive surgery; NACT = neoadjuvant chemotherapy; LN = lymph node.

FIGO Stage	Tumor Characteristics (LVSI, Invasion, Size)	Fertility-Sparing Procedures	Eligibility Criteria and Surgical Notes
**IA1 (≤3 mm)**	LVSI: absent or present (microinvasive ≤ 3 mm)	- **Conization** (coldknife cone or LEEP) with negative margins. - **Simple trachelectomy** if cone margins are inadequate.	- If LVSI(+): perform pelvic lymph node evaluation (SLN mapping or full PLND). - If cone margins positive: repeat excision or proceed to trachelectomy. - Place permanent cerclage during any trachelectomy.
**IA2 (3–5 mm)**	LVSI: absent or present (stromal invasion 3–5 mm)	- **Conization** + pelvic lymph node evaluation (SLN/PLND). - **Radical trachelectomy** (vaginal or abdominal/MIS) + pelvic LN dissection.	- Mandatory pelvic lymph node staging (SLN or full PLND). - Negative margins required; if cone margins positive, convert to trachelectomy. - Place permanent cerclage during trachelectomy.
**IB1 (≤2 cm)**	LVSI: absent or present (tumor ≤ 2 cm)	- **Conization or simple trachelectomy** (for very small, superficial LVSI– tumors). - **Radical trachelectomy** (vaginal or abdominal/MIS) + pelvic lymphadenectomy.	- Pelvic lymph node staging required (± paraaortic dissection based on risk). - LVSI (+) or deep invasion: favor radical trachelectomy. - Negative resection margins essential. - Place cerclage during trachelectomy.
**IB2 (2–4 cm)**	Tumor ~2–4 cm	- **Radical trachelectomy** (vaginal or abdominal) + pelvic (±paraaortic) LN dissection—select cases only (often after tumor NACT).	- Fertilitysparing *not standard* for tumors > 2 cm; high recurrence risk. - Thorough counseling on oncologic risk. - Mandatory pelvic ± paraaortic lymphadenectomy. - Place cerclage if trachelectomy performed.
**IB3 (>4 cm)**	Tumor > 4 cm	- **None**—fertility preservation contraindicated.	- No fertilitysparing surgery indicated; standard radical management required.

## Abbreviations

The following abbreviations are used in this manuscript:

ADK	Adenocarcinoma
ART	Abdominal Radical Trachelectomy
ASC	Adenosquamous Carcinoma
CCR	Concurrent Chemoradiotherapy
CRT	Chemoradiotherapy
EBRT	External Beam Radiation Therapy
ESGO	European Society of Gynaecological Oncology
FIGO	Fédération Internationale de Gynécologie et d’Obstétrique
HPV	Human Papillomavirus
IGBT	Image-Guided Brachytherapy
IMRT	Intensity-Modulated Radiation Therapy
LEEP	Loop Electrosurgical Excision Procedure
LN	Lymph Node
LND	Lymph Node Dissection
LVSI	Lymphovascular Space Invasion
MIS	Minimally Invasive Surgery
NACT	Neoadjuvant Chemotherapy
OT	Ovarian Transposition
PMA	Procreative Medical Assistance (e.g., IVF, IUI)
PLND	Pelvic Lymph Node Dissection
pCR	Pathological Complete Response
PR	Partial Response
PROM	Premature Rupture of Membranes
RRT	Robotic Radical Trachelectomy
SCC	Squamous Cell Carcinoma
SD	Stable Disease
SLN	Sentinel Lymph Node
SIR	Standardized Incidence Rate
TEP	Paclitaxel-Epirubicin-Cisplatin
TIP	Paclitaxel-Ifosfamide-Cisplatin
TLRT	Total Laparoscopic Radical Trachelectomy
TP	Paclitaxel-Cisplatin
VRT	Vaginal Radical Trachelectomy
3D	Three-Dimensional
